# Salivary Oxidative Stress Biomarkers as Predictors of Oral Health Risk Among Coffee-Drinking Smokers and Non-Smokers: Implications for Preventive Dentistry

**DOI:** 10.3290/j.ohpd.c_2710

**Published:** 2026-07-17

**Authors:** Poppy Andriany, Cut Fera Novita, Herwanda Herwanda, Munifah Abdat, Subhaini Jakfar, Basri A. Gani

**Affiliations:** a Poppy Andriany Dentist, Department of Dental Public Health, Dentistry Faculty, Universitas Syiah Kuala, Darussalam, Banda Aceh, Aceh, Indonesia. Study design, conducting the study, analysing the data, writing and reviewing the manuscript.; b Cut Fera Novita Dentist, Department of Dental Public Health, Dentistry Faculty, Universitas Syiah Kuala, Darussalam, Banda Aceh, Aceh, Indonesia. Study design, conducting the study, analysing the data, writing and reviewing the manuscript.; c Herwanda Herwanda Dentist, Department of Dental Public Health, Dentistry Faculty, Universitas Syiah Kuala, Darussalam, Banda Aceh, Aceh, Indonesia. Study design, conducting the study, analysing the data, writing and reviewing the manuscript.; d Munifah Abdat Dentist, Department of Dental Public Health, Dentistry Faculty, Universitas Syiah Kuala, Darussalam, Banda Aceh, Aceh, Indonesia. Study design, conducting the study, analysing the data, writing and reviewing the manuscript.; e Subhaini Jakfar Dentist, Department of Dental Material, Dentistry Faculty, Universitas Syiah Kuala, Darussalam, Banda Aceh, Aceh, Indonesia. Study design, conducting the study, analysing the data, writing and reviewing the manuscript.; f Basri A. Gani Dentist, Department of Oral Biology, Dentistry Faculty, Universitas Syiah Kuala, Darus-salam, Banda Aceh, Aceh, Indonesia. Study design, conducting the study, analysing the data, writing and reviewing the manuscript.

**Keywords:** oxidative stress, malondialdehyde, preventive dentistry, saliva, superoxide dismutase, student health

## Abstract

**Purpose:**

The goal of this study was to look at the link between smoking, drinking coffee, and salivary oxidative stress biomarkers malondialdehyde (MDA) and superoxide dismutase (SOD) as signs of oral health risk among university students in Aceh, Indonesia, and to see if they could be used as non-invasive screening tools in a clinical setting.

**Methods and Materials:**

There were three groups of students: active smokers, passive smokers, and non-smokers. We used validated questionnaires (Cronbach’s alpha = 0.872) to gather behavioural data. We collected unstimulated saliva in a controlled manner and then used enzyme-linked immunosorbent assay (ELISA) to analyse it. We also looked at the physicochemical properties of saliva. We used ANOVA, the Kruskal–Wallis test, and receiver operating characteristic (ROC) curve analysis to analyse the data.

**Results:**

Smokers had much higher MDA levels (4.1 ± 0.05 nmol/ml) and much lower SOD levels (0.72 ± 0.010 U/ml) than non-smokers (2.5 ± 0.01 nmol/ml; 1.20 ± 0.012 U/ml; *P* < 0.001). Drinking coffee by itself didn’t have a direct effect on oxidative stress, but when combined with smoking, it made the oxidative imbalance much worse. ROC analysis showed that the tests were very good at diagnosing (AUC = 0.88 for MDA; 0.84 for SOD).

**Conclusion:**

Salivary MDA and SOD are reliable, non-invasive markers that can detect early oxidative stress-related risk. It is a good idea to add saliva-based screening and culturally appropriate prevention strategies to campus health services. These results show that salivary biomarkers are useful in clinical settings for preventive dentistry.

Oxidative stress, a state characterised by an imbalance between the production of reactive oxygen species (ROS) and the body’s antioxidant defence mechanisms, is widely recognised as a central contributor to both oral and systemic diseases.^3^ These include periodontitis, oral squamous cell carcinoma, cardiovascular disease, and metabolic disorders such as diabetes mellitus and obesity-associated inflammation.^4^ Among young adults, particularly university students, lifestyle factors like cigarette smoking and coffee consumption are prevalent and frequently co-occur, potentially compounding oxidative burden at the cellular level.^24^

Smoking has been shown to influence periodontal disease progression and long-term treatment outcomes significantly.^28^ Cigarette smoke contains thousands of harmful chemicals, including free radicals and reactive aldehydes, that accelerate lipid peroxidation and oxidative damage to tissues.^35^ Simultaneously, coffee, while rich in antioxidants such as chlorogenic acids and polyphenols, may exhibit pro-oxidant effects when consumed in excess or in combination with smoking due to its caffeine content and metal-binding properties.^30^ While several studies have independently explored the oxidative impacts of smoking and caffeine,^11^ very few have examined their combined effect on salivary redox biomarkers, especially in student populations with distinct cultural habits, such as in Aceh, Indonesia, where black coffee and tobacco use are part of daily social norms.^34^ Recent advances highlight the critical role of host-response modulation and oxidative stress in periodontal disease pathogenesis, particularly among smokers and individuals with systemic conditions. Emerging evidence also supports the clinical utility of saliva as a non-invasive diagnostic medium for monitoring oral and systemic diseases.^9^

Saliva has become a dependable non-invasive diagnostic tool for assessing oral and systemic diseases.^10^ Saliva is increasingly acknowledged as a non-invasive, cost-effective biofluid for assessing oxidative status, providing benefits for screening extensive populations.^22^ Biomarkers like malondialdehyde (MDA), a byproduct of lipid peroxidation, and superoxide dismutase (SOD), a crucial enzymatic antioxidant, have demonstrated potential as indicators of oxidative stress in clinical and community contexts.^2^ Periodontal diseases are progressively comprehended via host-response mechanisms and biomarker-based diagnostics.^5^ The usefulness of measuring MDA and SOD for diagnosing combined effects, such as smoking and coffee consumption, hasn’t been thoroughly studied in Southeast Asia.^33^ Also, using saliva tests to assess oxidative stress in the community, especially among students, is still limited.^21^

This study directly supports the title by providing evidence that salivary oxidative stress biomarkers, specifically malondialdehyde (MDA) and SOD, can serve as early indicators of oral health risk in young adults who exhibit culturally embedded behaviours, such as smoking and habitual coffee consumption. Various biological fluids, including gingival crevicular fluid and saliva, have been explored for periodontal diagnostics.^6^ By addressing a critical research gap, this study is among the first to simultaneously analyse both oxidative and antioxidant biomarkers in relation to smoking and caffeine exposure among university students. The novelty lies in its dual-biomarker approach and the application of ROC (receiver operating characteristic) analysis, which adds diagnostic precision and supports the screening potential of saliva in community dental settings.

This study, conducted in Aceh, Indonesia, where traditional coffee and tobacco consumption are integral to daily life, presents a culturally contextualised model for predicting redox imbalance and its consequences for the onset of oral diseases, such as gingival inflammation, caries, and possible systemic effects. This study sought to assess the synergistic effects of smoking and coffee intake on salivary oxidative stress biomarkers (MDA and SOD) and to evaluate their clinical applicability as non-invasive indicators of oral health risk in young adults. The hypothesis proposed that individuals who both smoked and frequently consumed coffee would show increased oxidative stress and reduced antioxidant capacity, as indicated by salivary biomarkers.

The results corroborate this hypothesis, as demonstrated by quantitative biomarker levels and ROC performance. This investigation highlights the importance of saliva-based diagnostic tools in preventive dentistry, specifically supporting the use of early, non-invasive screening techniques for oral diseases linked to oxidative stress, particularly among at-risk adolescent groups. These findings support global efforts to include behavioural risk factors in preventive oral health programmes. They also help create screening tools that are both affordable and easy to use in universities and community health centres.

## METHODS AND MATERIALS

### Study Design and Population

This study was a cross-sectional observational study to evaluate the relationships among smoking habits, coffee consumption, and salivary oxidative stress biomarkers. A total of 90 university students were recruited and divided into three groups: active smokers, passive smokers, and non-smokers who consume coffee, with 30 subjects per group. Participants were selected using purposive sampling based on the following inclusion criteria: healthy systemic condition, aged 18–24 years, and not taking antibiotics or antioxidant supplements within the past 2 weeks. Subjects who declined participation or had underlying systemic diseases were excluded.

### Questionnaire Instrument and Validation

Data on lifestyle behaviours were collected using a semi-structured questionnaire covering smoking frequency and duration, coffee consumption patterns, and oral hygiene habits.^15^ The content of the questionnaire was validated by three experts in community dentistry and biomedical sciences. All items were deemed relevant. The instrument’s internal consistency was assessed using Cronbach’s Alpha, yielding α = 0.872, indicating high reliability.

### Saliva Collection and Storage Procedures

Unstimulated saliva samples were collected in the morning between 08:00 and 09:00 AM, after a minimum 90-min fasting period.^13^ Subjects were instructed to expectorate into sterile tubes for 5 min. The samples were centrifuged at 3000 rpm for 10 min to separate the supernatant, which was then stored at –20°C in labelled microcentrifuge tubes until further biomarker analysis.

### Assessment of Salivary Physical Characteristics

Several physical characteristics of saliva were assessed, including pH, flow rate, viscosity, and density. Salivary pH was measured using a digital pH meter immediately after collection. The flow rate was calculated by dividing the volume collected by time (ml/min). Viscosity was visually determined from fluid consistency in the collection tube, and density was measured using a precision scale in g/ml. All analyses were conducted under standardised laboratory conditions.

### Biomarker Analysis

This study examined oxidative stress indicators in saliva, focusing on malondialdehyde (MDA), a recognised marker of lipid peroxidation, and SOD, an internal antioxidant enzyme. The biomarkers were measured using enzyme-linked immunosorbent assay (ELISA) kits from Bioenzy™ (China) according to the manufacturer’s instructions. Each sample was analysed twice, and the results were reported in nmol/ml for MDA and U/ml for SOD.^16^ The ELISA kit offers an indirect evaluation of SOD activity-equivalent levels instead of absolute enzyme concentration, thus indicating the functional antioxidant capacity in saliva. To reduce the variability in salivary secretion, biomarker levels were standardised to the salivary flow rate (ml/min). This allowed for more precise, comparable measurements across individuals with different salivary outputs. In this study, the ELISA method was used to identify MDA–protein adducts, rather than free MDA. Due to MDA’s high reactivity and low molecular weight, it is predominantly quantified through TBARS or chromatographic techniques. Consequently, the results should be understood as indicative of protein modifications resulting from lipid peroxidation, rather than as a direct quantification of free MDA.

### Statistical Analysis

All data were analysed utilising SPSS version 25. The Kolmogorov–Smirnov test was employed to evaluate normality. Group disparities were assessed using one-way ANOVA or the Kruskal–Wallis test, as deemed appropriate. Pearson’s correlation was employed to assess relationships between variables. A multivariate linear regression model was used to assess the combined effects of smoking status, coffee consumption, and gender on MDA and SOD levels, presenting adjusted coefficients (β) for improved clarity. Furthermore, ROC curve analysis was performed to determine the diagnostic accuracy of the biomarkers.

### Ethical Approval

This study received ethical clearance from the Health Research Ethics Committee, Faculty of Dentistry, Universitas Syiah Kuala, with approval number 137/KE/FKGUSK/2025. All participants were given a full explanation of the study objectives and procedures, and written informed consent was obtained. Subject confidentiality was ensured through the use of anonymised data coding.

## RESULTS

Table 1 shows that 90 participants were evenly allocated by gender and smoking status. Almost 50% of individuals consumed coffee more than twice daily, with over one-third engaging in smoking while drinking coffee, and 30% exposed to secondhand smoke. The majority of participants maintained fundamental oral hygiene practices, although mouthwash use was minimal. Supplementary lifestyle factors, such as elevated sugar intake, inadequate water intake, and antioxidant use, may contribute to discrepancies in salivary oxidative stress biomarkers.

**Table 1:** Subject characteristics

Category	Sub-characteristics	N	Percentage (%)
Demographics	Male	45	50.0
	Female	45	50.0
Smoking status	Active smokers	30	33.3
	Passive smokers	30	33.3
	Non-smokers	30	33.3
Coffee consumption	Frequency > 2 times/day	42	46.7
	Frequency ≤ 2 times/day	48	53.3
	Smoking while drinking coffee	33	36.7
Exposure to cigarette smoke	Exposure at coffee shops	27	30.0
	No exposure in public places	63	70.0
Oral health behaviour	Regular mouthwash use	18	20.0
	Brushing teeth ≥ 2 times/day	60	66.7
	Complaints (dry mouth, canker sores, bad breath)	24	26.7
Lifestyle & nutrition	High sugar food consumption > 2 times/day	30	33.3
	Drinking < 1 litre of water/day	15	16.7
	Antioxidant supplement intake (vitamin C/E)	21	23.3
Data source: Author’s own research, 2025.			

The validation process showed that both the questionnaire and the biological samples were reliable, as shown in Table 2. The instrument exhibited substantial internal consistency (Cronbach’s alpha = 0.872), and saliva samples were collected and preserved under standardised conditions, ensuring stability without significant alterations in pH, volume, or appearance, thereby facilitating reliable MDA and SOD analysis.

**Table 2:** Validation of data and saliva samples

Aspect	Indicator	Result
Questionnaire instrument validation	Number of experts for content validation	3 experts (Community Dental Health and Biomedical)
	Content validation results	All items are deemed appropriate and relevant.
	Reliability test (Cronbach’s Alpha)	α = 0.872 (high reliability category)
Unstimulated saliva collection	Time of collection	08.00–09.00 AM (fasting ≥ 90 min)
	Average saliva volume	2.4 ml (range: 2–3 ml)
	Initial saliva pH	Neutral (6.8–7.2)
Sample storage procedure	Centrifugation procedure	3000 rpm for 10 min
	Storage temperature	–20°C (in microcentrifuge tubes)
Sample quality and stability check	pH and volume check after thawing	No significant changes (± 0.1 pH unit)
	Sample observation	No color change or precipitation


According to Table 3, active smokers drank the most coffee (2.7 times a day), usually while they were smoking. Passive smokers drank the second most coffee (2.1 times a day), and non-smokers drank the least (1.5 times a day). Most people liked unsweetened black Acehnese coffee and drank it at different times of day, indicating that it was part of their daily lives.

**Table 3:** Coffee consumption frequency by subject group

Subject group	Number of subjects	Average coffee intake (times/day)	Range	Additional notes
Active Smokers	30	2.7 times/day	1–5 times/day	60% consumed coffee while smoking
Passive Smokers	30	2.1 times/day	0–4 times/day	40% reported passive smoke exposure in coffee shop environments
Non-Smokers	30	1.5 times/day	0–3 times/day	70% drank coffee for study or alertness purposes
Notes: The majority of participants consumed black Acehnese coffee without sugar (75%), followed by instant sachet coffee (25%). Timing of coffee intake (Morning: 40%, Afternoon: 25%, Evening: 35%)				

Table 4 shows that active smokers had the lowest salivary pH, the slowest flow rate, and the thickest and most dense saliva compared with passive and non-smokers (*P* < 0.05). These results show that smoking changes the chemical and physical properties of saliva in a big way, which could make oral health more at risk.

**Table 4:** Saliva characteristics by subject group

Parameter	Active smokers (n = 30)	Passive smokers (n = 30)	Non-smokers (n = 30)	*P* value
Saliva pH (mean ± SD)	6.5 ± 0.2	6.8 ± 0.2	7.0 ± 0.1	< 0.001*
Salivary flow rate (ml/min)	0.2 ± 0.1	0.4 ± 0.1	0.6 ± 0.1	< 0.001*
Saliva viscosity	High in 73%	Normal in 87%	Normal in 100%	0.002**
Saliva density (g/ml)	1.03 ± 0.01	1.01 ± 0.01	1.00 ± 0.01	0.031*
* One-way ANOVA; ** Kruskal–Wallis test.				

Table 5 shows that the groups had different levels of salivary oxidative stress biomarkers. Active smokers had higher levels of MDA and lower levels of SOD. This means that smoking and drinking coffee regularly caused a lot of oxidative stress. Passive smokers had values that were in between, which shows the effects of being around secondhand smoke. On the other hand, non-smokers who drank coffee had the lowest levels of MDA and the highest levels of SOD. This suggests that coffee protects people who don’t smoke from the harmful effects of tobacco. These results suggest that smoking and coffee together are harmful, but coffee alone may help maintain antioxidant balance.

**Table 5:** Salivary biomarker profile (MDA and SOD) based on subject group with coffee consumption

Subject group	N	MDA (Mean ± SD, nmol/ml)	SOD (Mean ± SD, U/ml)	Subject description
Active Smokers	30	4.1 ± 0.05	0.72 ± 0.010	Smokes ≥1 cigarette/day; 60% drink coffee frequently (2–4×/day), mainly while smoking
Passive Smokers	30	3.2 ± 0.03	0.95 ± 0.08	Not active smokers but exposed to secondhand smoke at home or coffee shops; 40% drink coffee lightly (<2×/day)
Non-Smokers	30	2.5 ± 0.01	1.20 ± 0.012	Non-smokers; 70% regularly consume coffee (1–3×/day) mainly for studying or alertness
*P value		0.001	0.001	
* One-way ANOVA.				

**Fig 1:**
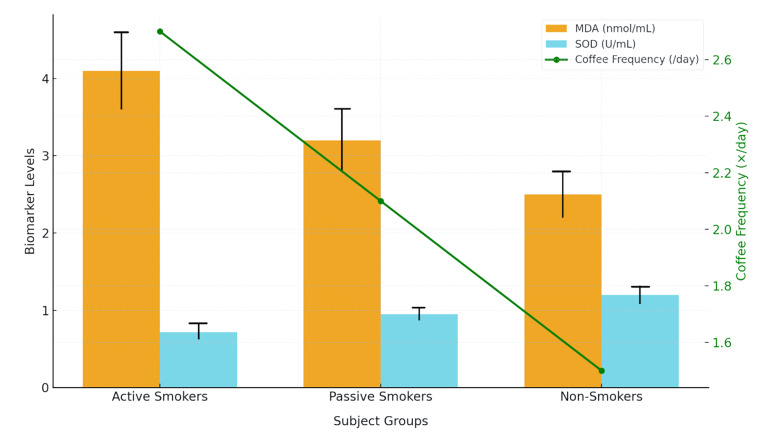
Comparison of salivary MDA (nmol/ml) and SOD (U/ml) levels among active smokers, passive smokers, and non-smokers, with a superimposed line graph indicating average coffee consumption frequency (times/day) for each group. Error bars represent standard deviation (SD).

Figure 1 shows that active smokers had the highest MDA and lowest SOD levels, which means they were under a lot of oxidative stress. Passive smokers were next, and non-smokers had the best biomarker profile. Drinking coffee alone didn’t have a clear effect on oxidative stress, but when combined with smoking, it increased MDA levels and decreased SOD levels much more, suggesting a synergistic effect.

**Fig 2a and b:**
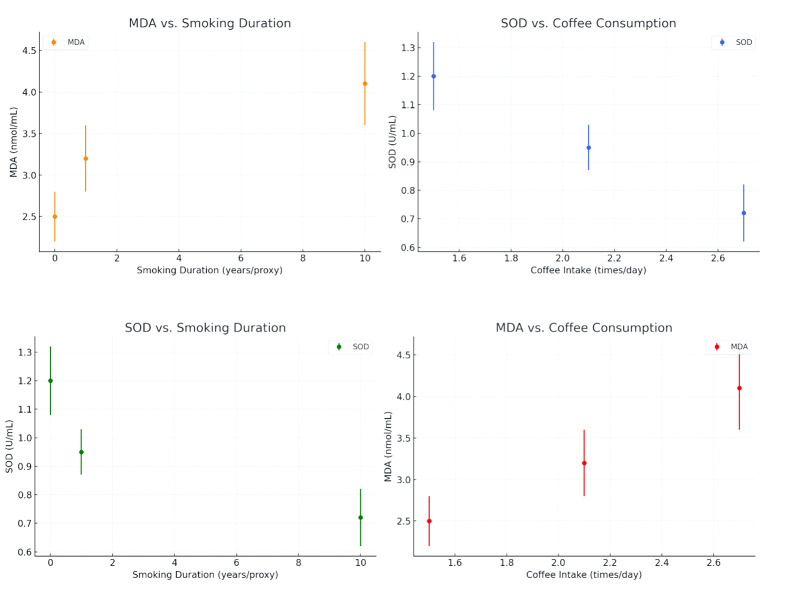
Relationship between lifestyle variables (duration of smoking and coffee intake) and salivary oxidative stress indicators (MDA and SOD) across subject groups. (a) A scatter plot that shows how MDA levels and smoking duration are related on the left and how SOD levels and coffee consumption are related on the right. (b) A scatter plot that shows how SOD levels and smoking duration are related on the left and how MDA levels and coffee consumption are related on the right. Each data point shows the mean ± standard deviation (SD) of salivary biomarkers for the group to which it belongs (active smokers, passive smokers, or non-smokers). The markers are colour-coded for clarity.

A correlation matrix showed that long-term smoking was strongly associated with higher MDA levels (r = 0.68, *P* < 0.001), and drinking coffee was strongly associated with lower SOD levels (r = –0.52, *P* < 0.01). This means that both how much you smoke and how you behave can cause oxidative imbalance. Figure 2 shows these connections even more clearly. It shows that MDA levels go up the longer you smoke, while SOD levels go down when you smoke or drink coffee. People who smoke had the most oxidative stress, while people who don’t smoke had the best profiles. The combined effect of smoking and drinking coffee suggests that they work together to make oxidative stress worse, rather than having separate, linear effects.

**Fig 3:**
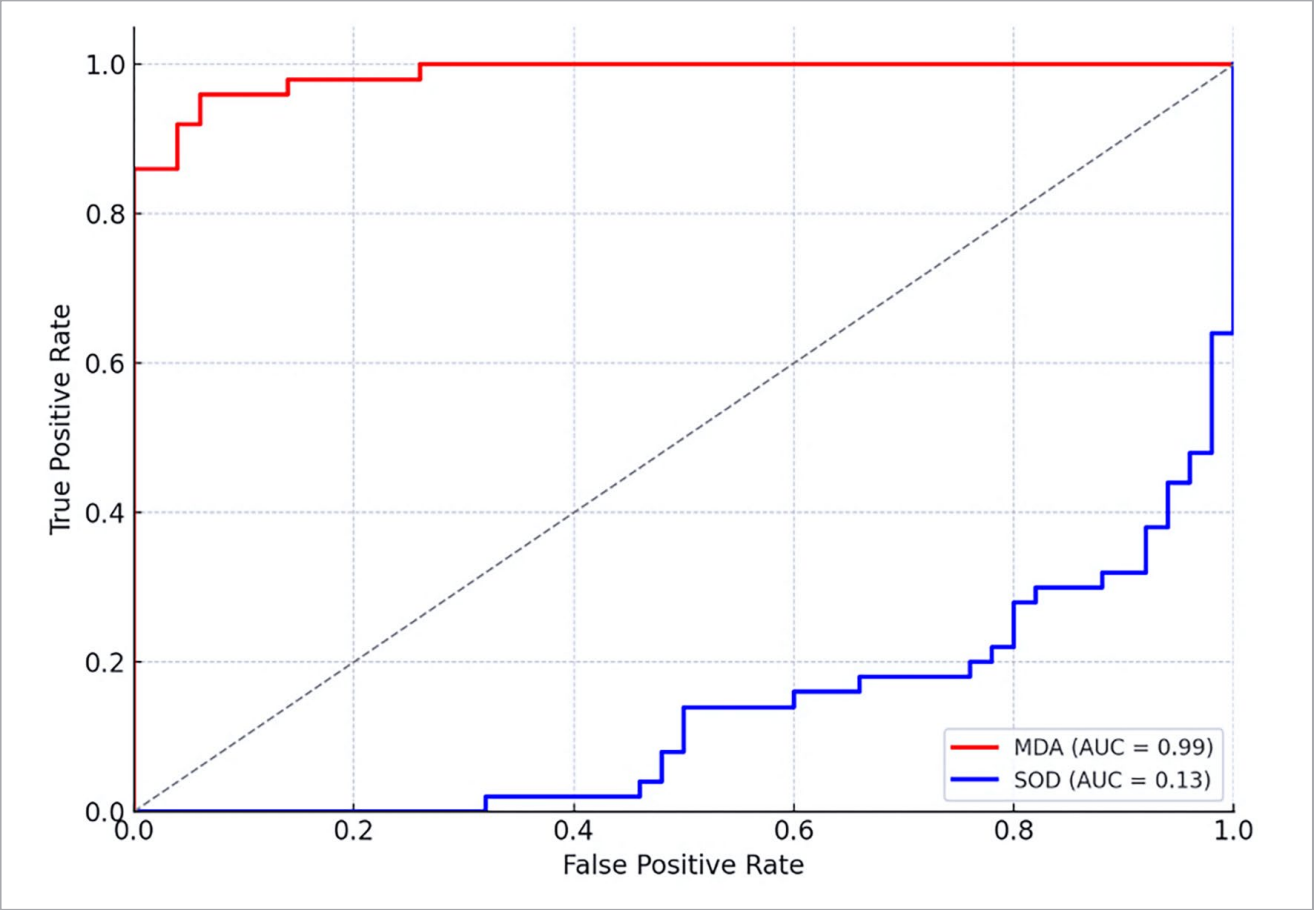
ROC curve of salivary biomarkers: MDA (AUC = 0.88) and SOD (AUC = 0.84) for predicting oxidative stress risk. Both biomarkers show strong discriminatory ability, with MDA showing slightly superior classification performance. The dashed diagonal line represents the no-discrimination line (AUC = 0.5).

Figure 3 shows that both MDA (AUC = 0.88) and SOD (AUC = 0.84) demonstrated high diagnostic accuracy in identifying oxidative stress. The best cut-off values for MDA were > 3.5 nmol/ml and for SOD were < 0.9 U/ml. This means these tests can serve as non-invasive biomarkers for early screening of conditions associated with oxidative stress.

Table 6 demonstrates that MDA and SOD effectively predict oral health risk associated with lifestyle factors. Conversely, the most favourable profiles were observed in non-smokers who consumed coffee, whereas smokers with high coffee intake exhibited the greatest oxidative stress. Given the high accuracy of saliva-based analysis, as evidenced by area under the curve values of 0.88 for MDA and 0.84 for SOD, this method provides a dependable, non-invasive approach for disease screening. These findings underscore the importance of lifestyle modifications and the development of culturally sensitive disease prevention strategies, such as incorporating salivary screening into community health initiatives.

**Table 6:** Summary of public health issues, salivary biomarker findings, and preventive dental implications

Issue	Key findings	Implications for oral health	Suggested preventive measures
Redox imbalance in smokers and coffee consumers	Active smokers with high coffee intake showed highest MDA (4.1 nmol/ml) and the lowest SOD (0.72 U/ml)	Elevated oxidative stress increases the risk of oral diseases (gingivitis, caries) and systemic conditions.	Implement university-based campaigns to reduce smoking and excessive caffeine consumption.
Diagnostic potential of salivary biomarkers	MDA (AUC = 0.88) and SOD (AUC = 0.84) demonstrate high diagnostic accuracy	Saliva is a reliable, non-invasive medium for early detection of oxidative stress	Integrate salivary biomarker screening into routine dental check-ups in campus health services
Impact of lifestyle behaviors on salivary redox status	Passive smokers show moderate alterations; non-smokers with moderate coffee intake show optimal biomarker levels	Modifiable lifestyle factors influence antioxidant balance and long-term oral health outcomes	Incorporate oral redox education into student health orientation and lifestyle intervention programs
Cultural influence: coffee and tobacco habits in Aceh 70% of non-smokers consume Acehnese coffee; 60% of smokers combine smoking and coffee		Cultural practices shape exposure patterns and biomarker expression, influencing disease risk	Develop culturally sensitive intervention strategies that respect local habits and traditions
Low awareness of oxidative stress (redox literacy)	65% of students lack knowledge about oxidative stress and its oral health impact	Low awareness delays preventive actions and increases disease susceptibility	Collaborate with student organizations to deliver culturally relevant health literacy campaigns
Underutilization of salivary diagnostics in student health services	No current implementation of salivary oxidative stress testing in university settings	Missed opportunity for early, non-invasive prevention of oral and metabolic diseases	Advocate for pilot programmes incorporating MDA/SOD analysis in annual student health screenings
Data source: Author’s own research, 2025.			

## DISCUSSION

This study shows a strong link between lifestyle habits, particularly smoking and coffee drinking, and salivary oxidative stress biomarkers. Active smokers had the highest MDA and the lowest SOD levels, which showed a severe redox imbalance. Non-smokers, on the other hand, had better profiles. Changes in the physicochemical properties of saliva that are significant also support the idea that these factors affect oral health. Saliva is a useful non-invasive screening tool because it has a high diagnostic accuracy (AUC: MDA 0.88; SOD 0.84).

People who smoked and drank coffee at the same time, were around secondhand smoke, and had bad habits were more likely to have oxidative stress. These results are in line with what we know about how tobacco exposure raises MDA and lowers SOD, which shows that redox homeostasis is out of whack.^18^ Coffee contains antioxidants like polyphenols, but too much or mixing it with smoking may shift its effects towards pro-oxidant, worsening oxidative damage.^19^ They also fit with new evidence that smoking speeds up the progression of periodontal disease and shows that salivary biomarkers can be used clinically for non-invasive diagnosis.^28^

The study maintained robust methodological rigour via validated instruments and standardised saliva handling (Table 2). The questionnaire demonstrated significant reliability (Cronbach’s alpha = 0.872) following expert validation, in accordance with epidemiological standards.^12^ Saliva was collected in a controlled setting (in the morning after a 90-min fast), and the samples stayed stable after processing and storage (–20°C). They had normal volume, pH, and appearance, indicating they were of the highest quality.^7^ This method is in line with current advice that saliva is a good, non-invasive way to measure oxidative stress.^25^ As a result, the results are trustworthy and back up the idea of using salivary MDA and SOD as useful biomarkers for screening and diagnosing oral diseases in the community.

People who smoked and drank coffee together were more likely to do both. Smokers drank coffee 2.7 times a day, which is a lot, and they often drank coffee while smoking, which may have increased oxidative stress. This fits with what we know about how nicotine and caffeine together increase ROS production.^31^ Most of the people who took part preferred unsweetened black Acehnese coffee, which is high in antioxidants like chlorogenic acid.^14^ However, smoking may make its protective effects less strong.^29^ So, the amount and timing of coffee drinking are both important for changing oxidative stress. This shows how important it is to look at groups of habits rather than just one.

Active smoking significantly altered the physical and chemical properties of saliva. This resulted in a lower pH (6.5), a slower flow rate (0.2 ml/min), and increased viscosity and density (*P* < 0.05). These changes indicate a more acidic and less healthy oral environment. As a result, saliva’s protective function is reduced, increasing the risk of cavities and gum disease.^27^ Changes in salivary profiles are linked to higher levels of MDA and lower levels of SOD activity. This means that oxidative stress is present and the body’s natural defences are not working as well.^20^ These results highlight the importance of using saliva tests and providing targeted health education to high-risk groups, such as young people who smoke.

The oxidative biomarker data in Table 5 reveal changes in lifestyle habits over time, specifically concerning smoking and coffee consumption. Smokers showed elevated malondialdehyde (MDA) levels and reduced SOD activity, suggesting increased oxidative stress. Conversely, non-smokers who consumed unsweetened black Acehnese coffee exhibited the most favourable profiles, suggesting that moderate consumption could offer some protection against oxidative stress. Passive smokers displayed intermediate values, which corroborate the detrimental health effects associated with secondhand smoke exposure. These findings align with previous research, which shows that various lifestyle risks can worsen oxidative stress.^23^ Coffee contains antioxidants, such as chlorogenic acid. However, smokers might not derive the same benefits because they are exposed to more free radicals. Measuring salivary MDA and SOD provides useful, non-invasive ways to assess oxidative stress driven by lifestyle choices. They could help with targeted interventions for young adults.

Figure 1 illustrates that coffee consumption alone does not markedly elevate oxidative stress; however, its impact becomes significant when coupled with smoking. People who don’t smoke and drink a lot of coffee had the best redox profile (high SOD and low MDA). On the other hand, people who smoke and drink a lot of coffee had higher MDA and lower SOD levels. This suggests a synergistic effect between coffee and smoking in worsening oxidative stress, which is in line with studies that show tobacco exposure increases free radical production and weakens antioxidant defences.^1^ Under some conditions, drinking too much coffee can also have pro-oxidant effects.^8^

Figure 2 shows a numerical link between how long someone smokes, how much coffee they drink, and markers of oxidative stress. As smoking lasted longer, MDA levels went up. At the same time, SOD levels went down when people drank more coffee and smoked for longer periods of time. The combination of these actions caused MDA to rise more quickly, showing how they work together to increase oxidative stress. These results are in line with other research that shows that being exposed to both nicotine and caffeine makes the redox imbalance worse.^1^ In general, the level of exposure and how long it lasts are both very important factors in oxidative stress.

The ROC analysis, as depicted in Figure 3, demonstrates that salivary malondialdehyde (MDA) and SOD exhibit strong diagnostic capabilities for oxidative stress, with areas under the curve (AUC) of 0.88 and 0.84, respectively. Consequently, these findings suggest that saliva is a viable, non-invasive, and cost-effective medium for assessing oxidative stress. The cut-off values identified (MDA > 3.5 nmol/ml; SOD < 0.9 U/ml) are useful for detecting cases early in both community and university settings, as in previous studies.^10^ Incorporating these biomarkers into campus health programmes could help identify people at risk earlier, especially those who engage in high-risk behaviours.^17^

Table 6 further emphasises public health issues, indicating that active smoking coupled with regular coffee intake substantially disturbs redox balance, elevating the risk of chronic diseases.^32^ On the other hand, non-smokers who drank unsweetened black coffee had better profiles, which could mean that it has antioxidant properties. These findings highlight the importance of targeted health education, culturally appropriate interventions, and saliva-based screening.^8^

In evaluating methodologies across studies, a critical assessment of the application of conventional thiobarbituric acid reactive substances (TBARS) or high-performance liquid chromatography (HPLC) techniques is essential. While the sample size of 90 participants was sufficient to detect intergroup disparities, the magnitude of these disparities may have posed challenges for conducting multivariate analyses. Consequently, the utilisation of larger cohorts is advisable for comprehensive modelling, thereby facilitating the consideration of genetic, dietary, and systemic influences. Furthermore, to improve the assessment of redox status, future studies should include biomarkers for oxidative stress and antioxidants. These could include advanced oxidation protein products (AOPP), catalase, and total antioxidant capacity (TAC). Salivary biomarkers, such as SOD and malondialdehyde (MDA), provide a non-invasive and efficient means to identify oral diseases associated with oxidative stress early. This approach streamlines the delivery of tailored and proactive dental services. To make periodontal care more cost-effective, we need to use preventive strategies that target modifiable risk factors.

## CONCLUSION

The present investigation suggests that salivary oxidative stress markers, specifically malondialdehyde (MDA) and SOD, may serve as indicators of oral health vulnerabilities in university students, contingent upon their smoking and coffee consumption habits. Students exhibiting high levels of both smoking and coffee intake demonstrated elevated MDA concentrations and diminished SOD levels. This means that their redox status is unbalanced and they are more likely to experience oral and systemic problems related to oxidative stress. Students who didn’t smoke but drank unsweetened Acehnese black coffee regularly, on the other hand, had more stable redox profiles. This means that drinking coffee by itself might not be bad for you, and it might even be pretty safe if you don’t smoke. These results show that saliva could be a simple, non-invasive way to detect oxidative stress early. Adding salivary screening to campus health services could be a useful way to move toward preventive care. Health interventions must be culturally appropriate, given that smoking and drinking coffee are common among students in Aceh. Raising awareness about oxidative stress and promoting healthier lifestyle choices may mitigate long-term oral health risks.

### Acknowledgements

The authors sincerely thank everyone who took part in the study, the laboratory team from the Faculty of Dentistry at Universitas Syiah Kuala, and the expert validators in community dentistry and biomedical sciences. We would like to thank the Research and Community Service Institute (LPPM) of Universitas Syiah Kuala for its financial support and help with this research.

#### Conflict of interest

The authors declare no conflict of interest related to this study.
